# Knowledge about self‐efficacy and outcomes in patients with heart failure and reduced ejection fraction

**DOI:** 10.1002/ejhf.2944

**Published:** 2023-07-26

**Authors:** Mingming Yang, Toru Kondo, Carly Adamson, Jawad H. Butt, William T. Abraham, Akshay S. Desai, Karola S. Jering, Lars Køber, Mikhail N. Kosiborod, Milton Packer, Jean L. Rouleau, Scott D. Solomon, Muthiah Vaduganathan, Michael R. Zile, Pardeep S. Jhund, John J.V. McMurray

**Affiliations:** ^1^ British Heart Foundation Cardiovascular Research Centre University of Glasgow Glasgow UK; ^2^ Department of Cardiology, Zhongda Hospital, School of Medicine Southeast University Nanjing China; ^3^ Department of Cardiology Nagoya University Graduate School of Medicine Nagoya Japan; ^4^ Department of Cardiology Copenhagen University Hospital Rigshospitalet Copenhagen Denmark; ^5^ Division of Cardiovascular Medicine The Ohio State University Ohio OH USA; ^6^ Cardiovascular Division Brigham and Women's Hospital, and Harvard Medical School Boston MA USA; ^7^ Saint Luke's Mid America Heart Institute and University of Missouri‐Kansas City Kansas MO USA; ^8^ Baylor Heart and Vascular Institute Baylor University Medical Center Dallas TX USA; ^9^ Institut de Cardiologie de Montréal Université de Montréal Montréal Canada; ^10^ Medical University of South Carolina and RHJ Department of Veterans Affairs Medical Center Charleston SC USA

**Keywords:** Kansas City Cardiomyopathy Questionnaire, Self‐efficacy, Self‐rated knowledge, Heart failure, Symptoms

## Abstract

**Aim:**

Although education in self‐management is thought to be an important aspect of the care of patients with heart failure, little is known about whether self‐rated knowledge of self‐management is associated with outcomes. The aim of this study was to assess the relationship between patient‐reported knowledge of self‐management and clinical outcomes in patients with heart failure and reduced ejection fraction (HFrEF).

**Methods and results:**

Using individual patient data from three recent clinical trials enrolling participants with HFrEF, we examined patient characteristics and clinical outcomes according to responses to the ‘self‐efficacy’ questions of the Kansas City Cardiomyopathy Questionnaire. One question quantifies patients' understanding of how to prevent heart failure exacerbations (‘prevention’ question) and the other how to manage complications when they arise (‘response’ question). Self‐reported answers from patients were pragmatically divided into: poor (do not understand at all, do not understand very well, somewhat understand), fair (mostly understand), and good (completely understand). Cox‐proportional hazard models were used to evaluate time‐to‐first occurrence of each endpoint, and negative binomial regression analysis was performed to compare the composite of total (first and repeat) heart failure hospitalizations and cardiovascular death across the above‐defined groups. Of patients (*n* = 17 629) completing the ‘prevention’ question, 4197 (23.8%), 6897 (39.1%), and 6535 (37.1%) patients had poor, fair, and good self‐rated knowledge, respectively. Of those completing the ‘response’ question (*n* = 17 637), 4033 (22.9%), 5463 (31.0%), and 8141 (46.2%) patients had poor, fair, and good self‐rated knowledge, respectively. For both questions, patients with ‘poor’ knowledge were older, more often female, and had a worse heart failure profile but similar treatment. The rates (95% confidence interval) per 100 person‐years for the primary composite outcome for ‘poor’, ‘moderate’ and ‘good’ self‐rated knowledge in answer to the ‘prevention’ question were 12.83 (12.11–13.60), 12.08 (11.53–12.65) and 11.55 (11.00–12.12), respectively, and for the ‘response’ question were 12.88 (12.13–13.67), 12.22 (11.60–12.86) and 11.56 (11.07–12.07), respectively. The lower event rates in patients with ‘good’ self‐rate knowledge were accounted for by lower rates of cardiovascular (and all‐cause) death and not hospitalization for worsening heart failure.

**Conclusions:**

Poor patient‐reported ‘self‐efficacy’ may be associated with higher rates of mortality. Evaluation of knowledge of ‘self‐efficacy’ may provide prognostic information and a guide to which patients may benefit from further education about self‐management.

## Graphical abstract

Patient‐reported knowledge of self‐care in heart failure (HF) with reduced ejection fraction. (*A*) Distribution of answers to the Kansas City Cardiomyopathy Questionnaire ‘prevention’ and ‘response’ questions. (*B*) Outcomes shown as rates per 100 person‐years according to category of patient‐reported knowledge of self‐care. In the right panel, the ‘Poor’ group includes ‘not at all’, ‘not very much’, and ‘somewhat’; ‘Fair’ includes ‘mostly’; ‘Good’ includes ‘completely. CV, cardiovascular.

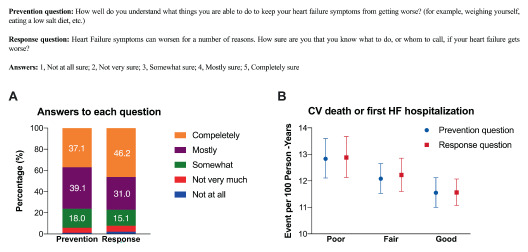

## Introduction

Over the past 20 years, the Kansas City Cardiomyopathy Questionnaire (KCCQ) has become the most commonly used tool to evaluate the health status of patients with heart failure. The KCCQ includes 23 items that map to seven domains: symptom frequency, symptom burden, symptom stability, physical limitations, social limitations, quality of life, and ‘self‐efficacy’.^1^, ^2^ The first two domains can be added to create the total symptom score and this can be combined with the physical limitation domain to construct the clinical summary score. The addition of the quality of life domain and social limitation domains to the clinical summary score creates the overall summary score. The various summary scores are often included as secondary endpoints in clinical trials. Notably, none of these scores utilizes the two questions that address ‘self‐efficacy’ (also described as ‘self‐care’ or ‘self‐management’) which are designed to quantify a patient's understanding of how to prevent heart failure exacerbations and manage complications when they arise. Unlike the other KCCQ domains (and derived summary scores), very little is published about these questions and their value. Of interest, the ‘self‐efficacy’ questions are reported to show a poor correlation with the other scales, suggesting independence between these items.^3^, ^4^, ^5^ Notably, the 12‐item version of the KCCQ does not include the ‘self‐efficacy’ questions.^6^ Nevertheless, contemporary practice and guidelines stress the importance of patient involvement in their own management and the value of self‐care.^7^, ^8^ For example, failure to restrict salt and fluid intake may reduce the efficacy of pharmacologic therapy and dietary non‐adherence, along with treatment non‐adherence, may increase the risk of decompensation.

Patient education efforts are rarely standardized, and accordingly, uptake of guidance by patients may be variable in practice; these ‘self‐efficacy’ questions may provide insight into the effectiveness of these efforts to enhance self‐care and provide opportunities for practice improvement in this area. Therefore, in this post hoc analysis, we examined the range of scores patients report for the KCCQ ‘self‐efficacy’ questions, patient characteristics related to these scores, and the association between scores and clinical outcomes among participants in three recent trials in patients with heart failure with reduced ejection fraction (HFrEF).

## Methods

### Trials and patients

In the present study, we conducted a post hoc analysis of pooled individual patient‐level data from three HFrEF trials (ATMOSPHERE, NCT00853658; PARADIGM‐HF, NCT01035255; and DAPA‐HF, NCT03036124) that collected the KCCQ ‘self‐efficacy’ domain questions. The designs, baseline characteristics, and primary results of these trials have been reported,^9^, ^10^, ^11^ and are summarized in online supplementary *Table* *S1*. All the trial protocols were approved by an ethics committee at each participating institution and written informed consent was provided by all patients.

### Patients' answers to the Kansas City Cardiomyopathy Questionnaire ‘self‐efficacy’ domain questions

One question (hereafter referred to as the ‘prevention’ question) asks ‘*How well do you understand what things you are able to do to keep your heart failure symptoms from getting worse (for example, regularly weighing yourself, eating a low salt diet, etc.)?*’, with a choice of five answers (do not understand at all, do not understand very well, somewhat understand, mostly understand, or completely understand). The other question (hereafter referred to as the ‘response’ question) asks ‘*Heart failure symptoms can worsen for a number of reasons. How sure are you that you know what to do or whom to call, if your heart failure gets worse?*’, with a choice of five answers (not at all sure, not very sure, somewhat sure, mostly sure, or completely sure) (online supplementary *Table* *S2*). Based on their self‐reported answers, patients were pragmatically divided into the following groups: poor (do not understand at all, do not understand very well, somewhat understand), fair (mostly understand), and good (completely understand) to ensure a reasonable distribution of numbers across categories for statistical analysis (e.g. there were very few patients in the ‘do not understand at all’ category).

### Clinical outcomes

The original primary outcomes for each trial can be found in online supplementary *Table* *S1*. In the present analysis, we examined the composite of time to a first hospitalization for heart failure or cardiovascular death, as well as the components of this composite. We also analysed the occurrence of death from any cause, as well as the composite of total (first and repeat) heart failure hospitalizations and cardiovascular death. All the outcomes were adjudicated by endpoint committees as indicated in the original trial reports.

### Statistical analysis

Baseline characteristics are presented as means with standard deviation, medians with interquartile ranges, or frequencies with percentages as appropriate. The incidence of each outcome is reported as a rate per 100 patient‐years of follow‐up. The time‐to‐first occurrence of each endpoint was evaluated using the Kaplan–Meier estimator and Cox proportional hazards models. Negative binomial regression analysis was performed to compare the composite of total (first and repeat) heart failure hospitalizations and cardiovascular death across the above‐defined groups, based on self‐reported answers. In addition, we also reported the hazard ratios (HR) and incidence rate ratios (IRR) from models adjusted for treatment arm, region, age, sex, heart rate, systolic blood pressure, body mass index, New York Heart Association (NYHA) functional class, left ventricular ejection fraction, estimated glomerular filtration rate, N‐terminal pro‐B‐type natriuretic peptide (NT‐proBNP), prior hospitalization for heart failure, atrial fibrillation, ischaemic aetiology, history of myocardial infarction, history of diabetes mellitus, and stroke. The missing indicator method was applied to impute data on NT‐proBNP values.^12^

The analysis was conducted using Stata/SE version 17.0 (Stata Corp, College Station, TX, USA). A significance level of 0.05 was considered to be statistically significant.

## Results

Among the 20 159 patients enrolled in the three HFrEF trials, a total of 17 629 (87.4%) patients answered the ‘prevention’ question and 17 637 (87.5%) patients answered the ‘response’ question (data for the individual trials can be found in online supplementary *Table* *S3*). For the ‘prevention’ question, patient self‐rated knowledge was poor, fair, and good in 4197 (23.8%), 6897 (39.1%), and 6535 (37.1%) patients, respectively (*Table* *1* and online supplementary *Figure* *S1*). For the ‘response’ question, these numbers/proportions were 4033 (22.9%), 5463 (31.0%), and 8141 (46.2%), respectively (*Table* *1* and online supplementary *Figure* *S1*). The number of patients in each response category for each question in the individual trials can be found in online supplementary *Table* *S4*.

**Table 1:** Baseline characteristics according to the answers to the Kansas City Cardiomyopathy Questionnaire self‐care questions in patients with heart failure and reduced ejection fraction

	KCCQ ‘prevention’ question			KCCQ ‘response’ question		
	Poor	Fair	Good	Poor	Fair	Good
*n* (%)	4197 (23.8)	6897 (39.1)	6535 (37.1)	4033 (22.9)	5463 (31.0)	8141 (46.2)
**Demographic characteristics**						
Age, years	65.5 ± 11.3	64.3 ± 11.1	64.0 ± 11.2	65.5 ± 11.3	64.6 ± 11.0	63.9 ± 11.2
Age >70 years	1537 (36.6)	2161 (31.3)	1976 (30.2)	1463 (36.3)	1763 (32.3)	2450 (30.1)
Sex						
Female	1093 (26.0)	1413 (20.5)	1321 (20.2)	1065 (26.4)	1149 (21.0)	1614 (19.8)
Male	3104 (74.0)	5484 (79.5)	5214 (79.8)	2968 (73.6)	4314 (79.0)	6527 (80.2)
Region						
North America	189 (4.5)	456 (6.6)	775 (11.9)	197 (4.9)	345 (6.3)	878 (10.8)
Latin America^a^	568 (13.5)	1062 (15.4)	1274 (19.5)	634 (15.7)	893 (16.3)	1374 (16.9)
Western Europe	877 (20.9)	1694 (24.6)	1734 (26.5)	929 (23.0)	1341 (24.5)	2041 (25.1)
Eastern Europe^b^	1849 (44.1)	2620 (38.0)	1780 (27.2)	1677 (41.6)	2058 (37.7)	2518 (30.9)
Asia/Pacific and other	714 (17.0)	1065 (15.4)	972 (14.9)	596 (14.8)	826 (15.1)	1330 (16.3)
Race						
White	3137 (74.7)	5157 (74.8)	4831 (73.9)	3040 (75.4)	4079 (74.7)	6016 (73.9)
Black	185 (4.4)	246 (3.6)	271 (4.1)	189 (4.7)	182 (3.3)	330 (4.1)
Asian	689 (16.4)	1033 (15.0)	958 (14.7)	580 (14.4)	804 (14.7)	1297 (15.9)
Others	186 (4.4)	461 (6.7)	475 (7.3)	224 (5.6)	398 (7.3)	498 (6.1)
SBP, mmHg	124.0 ± 16.6	122.8 ± 16.5	121.5 ± 16.5	123.2 ± 16.6	123.5 ± 16.5	121.8 ± 16.5
SBP <110 mmHg	767 (18.3)	1400 (20.3)	1538 (23.5)	803 (19.9)	1042 (19.1)	1862 (22.9)
HR, bpm	72.8 ± 12.3	71.7 ± 11.9	71.0 ± 12.1	72.4 ± 12.4	71.9 ± 11.8	71.3 ± 12.1
BMI, kg/m^2^	27.4 (24.1–31.1)	27.5 (24.5–31.3)	27.8 (24.6–31.4)	27.2 (24.3–31.2)	27.4 (24.4–31.1)	27.8 (24.6–31.5)
Weight category						
<18.5	82 (2.0)	97 (1.4)	77 (1.2)	67 (1.7)	83 (1.5)	106 (1.3)
18.5–25.0	1158 (27.6)	1795 (26.1)	1680 (25.8)	1088 (27.0)	1436 (26.3)	2110 (26.0)
25.0–30	1558 (37.1)	2670 (38.8)	2535 (38.9)	1538 (38.2)	2136 (39.1)	3093 (38.0)
≥30.0	1397 (33.3)	2328 (33.8)	2231 (34.2)	1336 (33.2)	1802 (33.0)	2821 (34.7)
**Comorbidities**						
Atrial fibrillation (history)	1714 (40.8)	2596 (37.6)	2346 (35.9)	1657 (41.1)	2083 (38.1)	2920 (35.9)
Hypertension	3047 (72.6)	4869 (70.6)	4442 (68.0)	2942 (72.9)	3931 (72.0)	5490 (67.4)
Angina pectoris	1345 (32.0)	1821 (26.4)	1516 (23.2)	1247 (30.9)	1486 (27.2)	1954 (24.0)
MI	1799 (42.9)	3005 (43.6)	2864 (43.8)	1698 (42.1)	2357 (43.1)	3615 (44.4)
Prior PCI/CABG	1277 (30.4)	2377 (34.5)	2411 (36.9)	1220 (30.3)	1820 (33.3)	3031 (37.2)
Stroke	401 (9.6)	554 (8.0)	518 (7.9)	393 (9.7)	466 (8.5)	614 (7.5)
COPD	604 (14.4)	871 (12.6)	782 (12.0)	556 (13.8)	717 (13.1)	984 (12.1)
Diabetes mellitus	1383 (33.0)	2389 (34.6)	2226 (34.1)	1355 (33.6)	1856 (34.0)	2793 (34.3)
Anaemia^c^	935 (22.7)	1466 (21.7)	1394 (21.7)	927 (23.4)	1182 (22.1)	1687 (21.1)
Current smoker	618 (14.7)	1010 (14.6)	857 (13.1)	540 (13.4)	776 (14.2)	1172 (14.4)
**HF characteristics and investigations**						
Ischaemic aetiology	2471 (58.9)	4018 (58.3)	3763 (57.6)	2342 (58.1)	3177 (58.2)	4740 (58.2)
Previous hospitalization for HF	2461 (58.6)	4065 (58.9)	3771 (57.7)	2344 (58.1)	3197 (58.5)	4760 (58.5)
NYHA class III/IV	1756 (41.8)	2282 (33.1)	1559 (23.9)	1682 (41.7)	1890 (34.6)	2028 (24.9)
KCCQ clinical summary score	65.0 ± 20.5	74.4 ± 18.6	79.6 ± 18.6	64.5 ± 20.6	73.8 ± 18.7	79.0 ± 18.5
Signs of congestion						
Dyspnoea on effort	2774 (90.6)	4750 (87.1)	4197 (84.0)	2625 (90.1)	3801 (88.0)	5301 (84.3)
Dyspnoea at rest	238 (7.8)	193 (3.5)	132 (2.6)	221 (7.6)	180 (4.2)	162 (2.6)
Orthopnoea	234 (7.6)	349 (6.4)	253 (5.1)	244 (8.4)	297 (6.9)	295 (4.7)
PND	261 (8.5)	255 (4.7)	172 (3.4)	260 (8.9)	232 (5.4)	196 (3.1)
Fatigue	1920 (62.7)	2819 (51.7)	2297 (45.9)	1803 (61.9)	2296 (53.2)	2943 (46.8)
Oedema	886 (28.9)	1116 (20.5)	903 (18.1)	850 (29.2)	931 (21.6)	1127 (17.9)
S3 gallop	278 (9.1)	454 (8.3)	390 (7.8)	260 (8.9)	400 (9.3)	461 (7.3)
JVD	370 (12.1)	464 (8.5)	431 (8.6)	350 (12.0)	426 (9.9)	490 (7.8)
Rales	405 (13.2)	450 (8.3)	366 (7.3)	383 (13.2)	392 (9.1)	446 (7.1)
ECG findings and NT‐proBNP						
Atrial fibrillation/flutter	1216 (29.2)	1741 (25.5)	1485 (22.9)	1155 (28.9)	1420 (26.2)	1871 (23.2)
NT‐proBNP, pg/ml	1512 (815–2921)	1423 (794–2691)	1374 (782–2608)	1536 (816–2928)	1452 (807–2821)	1357 (775–2556)
Atrial fibrillation/flutter^d^	1864 (1151–3361)	1851 (1114–3209)	1876 (1168–3222)	1922 (1151–3345)	1821 (1137–3209)	1864 (1154–3222)
No atrial fibrillation/flutter^d^	1340 (720–2704)	1253 (721–2437)	1240 (717–2397)	1346 (728–2720)	1289 (724–2580)	1215 (712–2314)
LVEF and other laboratory investigations						
LVEF, %	30.5 ± 6.0	29.6 ± 6.1	29.1 ± 6.4	30.2 ± 6.2	29.7 ± 6.1	29.3 ± 6.3
Haemoglobin, g/L	138.0 (127.0–148.0)	139.0 (129.0–150.0)	139.0 (128.0–149.0)	137.0 (127.0–148.0)	139.0 (128.0–149.0)	139.0 (129.0–150.0)
Creatinine, μmol/L	93.0 (79.0–112.0)	94.0 (80.0–111.0)	95.0 (80.0–113.2)	93.2 (79.0–112.0)	94.0 (80.0–111.2)	94.2 (80.0–113.0)
eGFR, ml/min/1.73 m^2^	67.0 (54.0–81.0)	68.0 (55.0–82.0)	67.0 (54.0–81.0)	67.0 (54.0–81.0)	68.0 (55.0–82.0)	68.0 (54.0–82.0)
eGFR <60 ml/min/1.73 m^2^	1467 (35.0)	2304 (33.4)	2272 (34.8)	1432 (35.5)	1825 (33.4)	2787 (34.3)
**Medication and other interventions**						
Diuretics	3584 (85.4)	5697 (82.6)	5456 (83.5)	3470 (86.0)	4567 (83.6)	6707 (82.4)
Loop	3194 (76.1)	5184 (75.2)	5006 (76.6)	3121 (77.4)	4167 (76.3)	6102 (75.0)
Thiazides	389 (9.3)	483 (7.0)	401 (6.1)	336 (8.3)	414 (7.6)	524 (6.4)
Digitalis	1174 (28.0)	1914 (27.8)	1688 (25.8)	1096 (27.2)	1511 (27.7)	2171 (26.7)
Beta‐blocker	3907 (93.1)	6476 (93.9)	6191 (94.7)	3756 (93.1)	5124 (93.8)	7701 (94.6)
MRA	2249 (53.6)	3766 (54.6)	3556 (54.4)	2210 (54.8)	2958 (54.1)	4405 (54.1)
ACEI/ARB/ARNI	4141 (98.7)	6813 (98.8)	6437 (98.5)	3970 (98.4)	5407 (99.0)	8022 (98.5)
ICD^e^	568 (13.5)	1268 (18.4)	1512 (23.1)	581 (14.4)	959 (17.6)	1811 (22.2)
CRT‐P or CRT‐D	224 (5.3)	460 (6.7)	552 (8.4)	219 (5.4)	370 (6.8)	646 (7.9)

Data are presented as mean ± standard deviation, median (interquartile range) for continuous measures, or *n* (%) for categorical measures.

ACEI, angiotensin‐converting enzyme inhibitor; ARB, angiotensin receptor blocker; ARNI, angiotensin receptor–neprilysin inhibitor; BMI, body mass index; CABG, coronary artery bypass grafting; COPD, chronic obstructive pulmonary disease; CRT‐D, cardiac resynchronization therapy with defibrillator; CRT‐P, cardiac resynchronization therapy with pacemaker; ECG, electrocardiogram; eGFR, estimated glomerular filtration rate; HF, heart failure; HR, heart rate; ICD, implantable cardioverter defibrillator; JVD, jugular venous distension; KCCQ, Kansas City Cardiomyopathy Questionnaire; LVEF, left ventricular ejection fraction; MI, myocardial infarction; MRA, mineralocorticoid receptor antagonist; NT‐proBNP, N‐terminal pro‐B‐type natriuretic peptide; NYHA, New York Heart Association; PCI, percutaneous coronary intervention; PND, paroxysmal nocturnal dyspnoea; SBP, systolic blood pressure.

^a^ Including Central America.

^b^ Including Central Europe and Russia.

^c^ Haemoglobin <130 g/L for males and <120 g/L for females.

^d^ Based on ECG.

^e^ Including CRT‐D.

### Patient characteristics according to self‐rated knowledge

For both questions, patients reporting ‘good’ compared to ‘poor’ knowledge were younger, more often male and from North and Latin America, and less often from Central/Eastern Europe (with little difference in the proportion of patients from Western Europe and Asia across the knowledge categories) (*Table* *1*; online supplementary *Figures* *S2*–*S4*). Several comorbidities including hypertension, atrial fibrillation, prior stroke, and COPD were less common in patients reporting ‘good’ compared to ‘poor’ knowledge and NYHA class and KCCQ clinical summary score were better in patients with ‘good’ knowledge. In keeping with this, patients with ‘good’ compared to ‘poor’ knowledge had fewer symptoms and signs of heart failure and lower average NT‐proBNP levels. However, the prevalence of prior hospitalization for heart failure did not differ across the self‐rated knowledge groups and the use of pharmacological therapy, including renin–angiotensin system inhibitors, beta‐blockers, and mineralocorticoid receptor antagonists was broadly similar in each knowledge category (although device therapy was more commonly used in patients with ‘good’ self‐rated knowledge). Blood pressure and heart rate were lower in patients reporting ‘good’ compared to ‘poor’ knowledge.

A comparison of patients answering and not answering the self‐efficacy questions is shown in online supplementary *Table* *S5*.

### Clinical outcomes according to self‐rated knowledge at baseline

**Figure 1:**
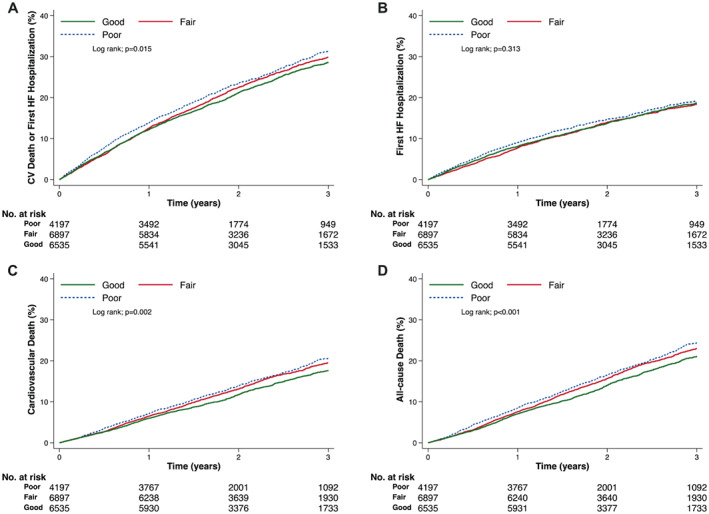
Cumulative incidence of outcomes according to answers to the ‘prevention’ self‐care Kansas City Cardiomyopathy Questionnaire question in patients with heart failure (HF) and reduced ejection fraction. (*A*) Cardiovascular (CV) death or first HF hospitalization; (*B*) first HF hospitalization; (*C*) CV death; (*D*) all‐cause death.

**Figure 2:**
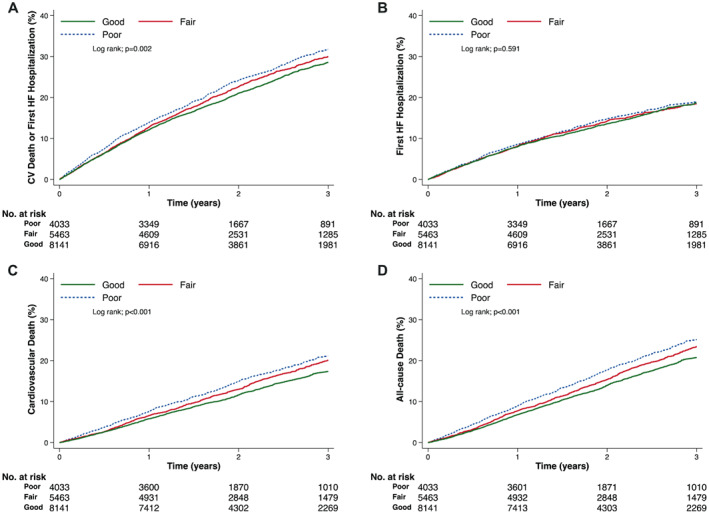
Cumulative incidence of outcomes according to answers to the ‘response’ self‐care Kansas City Cardiomyopathy Questionnaire question in patients with heart failure (HF) and reduced ejection fraction. (*A*) Cardiovascular (CV) death or first HF hospitalization; (*B*) first HF hospitalization; (*C*) CV death; (*D*) all‐cause death.

For each question, patients reporting ‘good’ compared to ‘poor’ knowledge generally had better outcomes (*Table* *2*, *Figures* *1* and *2*, and *Graphical Abstract*). This was most striking for all‐cause mortality where the unadjusted HR for ‘good’ compared to ‘poor’ knowledge in answer to the ‘response’ question was 0.82 (0.76–0.90) (*p* < 0.001). Even after comprehensive adjustment for recognized prognostic variables, including NT‐proBNP the adjusted HR (aHR) was 0.87 (0.80–0.95) (*p* = 0.002). The corresponding unadjusted and adjusted HRs for the ‘prevention’ question were 0.85 (0.78–0.93) (*p* < 0.001) and 0.90 (0.83–0.99) (*p* = 0.023), respectively. A broadly similar picture was seen when patients who reported ‘poor’ knowledge in response to both questions were compared to patients reporting ‘good’ knowledge in response to both questions (online supplementary *Table* *S6*).

**Table 2:** Clinical outcomes according to the answers to the Kansas City Cardiomyopathy Questionnaire self‐care questions in patients with heart failure and reduced ejection fraction

	KCCQ ‘prevention’ question			KCCQ ‘response’ question		
	Poor	Fair	Good	Poor	Fair	Good
*n* (%)	4197 (23.8)	6897 (39.1)	6535 (37.1)	4033 (22.9)	5463 (31.0)	8141 (46.2)
Primary composite outcome						
No. of events (%)	1132 (27.0)	1814 (26.3)	1632 (25.0)	1085 (26.9)	1446 (26.5)	2050 (25.2)
Event rate per 100 person‐years (95% CI)	12.83 (12.11–13.60)	12.08 (11.53–12.65)	11.55 (11.00–12.12)	12.88 (12.13–13.67)	12.22 (11.60–12.86)	11.56 (11.07–12.07)
Unadjusted HR (95% CI)	1.00 (Ref.)	0.94 (0.88–1.02)	0.90 (0.83–0.97)	1.00 (Ref.)	0.95 (0.88–1.03)	0.90 (0.84–0.97)
*p*‐value		0.121	0.007		0.213	0.005
Adjusted HR (95% CI)^a^	1.00 (Ref.)	0.96 (0.89–1.04)	0.94 (0.87–1.02)	1.00 (Ref.)	0.98 (0.90–1.06)	0.95 (0.88–1.02)
*p*‐value		0.317	0.146		0.587	0.161
First HF hospitalization						
No. of events (%)	652 (15.5)	1042 (15.1)	998 (15.3)	603 (15.0)	838 (15.3)	1252 (15.4)
Event rate per 100 person‐years (95% CI)	7.39 (6.84–7.98)	6.94 (6.53–7.37)	7.06 (6.64–7.51)	7.16 (6.61–7.75)	7.08 (6.62–7.58)	7.06 (6.68–7.46)
Unadjusted HR (95% CI)	1.00 (Ref.)	0.94 (0.85–1.04)	0.96 (0.87–1.06)	1.00 (Ref.)	0.99 (0.89–1.10)	0.99 (0.90–1.09)
*p*‐value		0.226	0.384		0.898	0.854
Adjusted HR (95% CI)^a^	1.00 (Ref.)	0.94 (0.85–1.04)	0.97 (0.87–1.07)	1.00 (Ref.)	1.02 (0.92–1.13)	1.02 (0.92–1.12)
*p*‐value		0.229	0.496		0.709	0.719
CV death						
No. of events (%)	736 (17.5)	1174 (17.0)	993 (15.2)	715 (17.7)	937 (17.2)	1253 (15.4)
Event rate per 100 person‐years (95% CI)	7.66 (7.12–8.23)	7.20 (6.80–7.62)	6.52 (6.13–6.9)4	7.85 (7.29–8.44)	7.29 (6.84–7.77)	6.52 (6.17–6.89)
Unadjusted HR (95% CI)	1.00 (Ref.)	0.94 (0.86–1.03)	0.85 (0.77–0.94)	1.00 (Ref.)	0.93 (0.84–1.02)	0.83 (0.76–0.91)
*p*‐value		0.172	0.001		0.126	<0.001
Adjusted HR (95% CI)^a^	1.00 (Ref.)	0.98 (0.89–1.07)	0.92 (0.84–1.02)	1.00 (Ref.)	0.95 (0.86–1.05)	0.89 (0.81–0.97)
*p*‐value		0.645	0.118		0.337	0.013
All‐cause death						
No. of events (%)	891 (21.2)	1416 (20.5)	1201 (18.4)	871 (21.6)	1120 (20.5)	1519 (18.7)
Event rate per 100 person‐years (95% CI)	9.27 (8.68–9.90)	8.68 (8.24–9.14)	7.89 (7.45–8.35)	9.56 (8.94–10.21)	8.71 (8.21–9.24)	7.91 (7.52–8.31)
Unadjusted HR (95% CI)	1.00 (Ref.)	0.93 (0.86–1.02)	0.85 (0.78–0.93)	1.00 (Ref.)	0.91 (0.83–0.99)	0.82 (0.76–0.90)
*p*‐value		0.107	<0.001		0.035	<0.001
Adjusted HR (95% CI)^a^	1.00 (Ref.)	0.97 (0.89–1.05)	0.90 (0.83–0.99)	1.00 (Ref.)	0.93 (0.85–1.02)	0.87 (0.80–0.95)
*p*‐value		0.420	0.023		0.114	0.002
Recurrent HF hospitalization/CV death						
No. of events	1811	2936	2665	1703	2327	3386
Event rate per 100 person‐years (95% CI)	18.84 (17.61–20.16)	17.99 (17.03–19.01)	17.50 (16.48–18.59)	18.69 (17.41–20.06)	18.11 (17.04–19.24)	17.63 (16.71–18.59)
Unadjusted IRR (95% CI)	1.00 (Ref.)	0.92 (0.83–1.02)	0.90 (0.82–1.00)	1.00 (Ref.)	0.91 (0.82–1.01)	0.86 (0.78–0.96)
*p*‐value		0.114	0.057		0.089	0.005
Adjusted IRR (95% CI)^a^	1.00 (Ref.)	0.96 (0.87–1.05)	0.95 (0.86–1.05)	1.00 (Ref.)	0.93 (0.84–1.03)	0.92 (0.83–1.01)
*p*‐value		0.365	0.336		0.142	0.066

CI, confidence interval; CV, cardiovascular; HF, heart failure; HR, hazard ratio; IRR, incidence rate ratio; KCCQ, Kansas City Cardiomyopathy Questionnaire.

^a^ Adjusted model has been adjusted for region, treatment arm, age, sex, heart rate, systolic blood pressure, body mass index, left ventricular ejection fraction, estimated glomerular filtration rate, N‐terminal pro‐B‐type natriuretic peptide, prior hospitalization for HF, atrial fibrillation, ischaemic aetiology, myocardial infarction, diabetes mellitus, and stroke.

A comparison of outcomes in patients answering and not answering the self‐efficacy questions is shown in online supplementary *Table* *S7*. Analyses using the self‐efficacy score showed a similar pattern to our original analysis (online supplementary *Table* *S8*).

## Discussion

Across three clinical trials enrolling 20 159 patients with HFrEF, nearly a quarter of enrolled patients reported poor self‐rated knowledge about ‘self‐efficacy’, indicating a perceived lack of awareness about how to self‐manage their condition. Although doubts have been expressed about the usefulness of the KCCQ ‘self‐efficacy’ questions, we found these questions seemed to be associated with mortality in patients with HFrEF.^3^, ^4^, ^5^ These data suggest that despite their simplicity, the KCCQ ‘self‐efficacy’ questions may be a relevant barometer of the success of patient education efforts with implications for subsequent prognosis.

Self‐rated knowledge about ‘self‐efficacy’ was also associated with differences in patient profiles. Specifically, patients who reported their knowledge as ‘poor’ had worse symptoms and signs, worse NYHA functional class, and higher NT‐proBNP levels. Adjustment for these differences attenuated the differences in mortality between patients with ‘good’ compared to ‘poor’ knowledge. However, whether this adjustment was appropriate is open to question given the potential ‘chicken or egg’ relationship, i.e. is worse overall heart failure status due to poor patient understanding and response to symptoms and signs, or does their worse overall condition make patients feel they do not understand their illness, how to self‐manage it, and when to seek help?

Importantly, greater self‐rated knowledge was more closely associated with fatal outcomes and rates of hospitalization for heart failure were not lower in patients with greater self‐reported knowledge. However, here too the relationship between self‐knowledge and the outcome is potentially confounded as seeking help in response to a change in clinical status may have resulted in hospital admission.

Notably, there was little difference in the use of pharmacologic therapies across the range of self‐rated knowledge about ‘self‐efficacy’ although, interestingly, both blood pressure and heart rate were lower in patients reporting ‘good’ compared to ‘poor’ knowledge, perhaps reflecting better adherence in those with ‘good’ knowledge.

The question is whether these findings are credible. At face value, it seems remarkable that the answers to two simple questions could provide prognostic information. Unfortunately, it is hard to corroborate these data as there are few other instruments that measure ‘self‐efficacy’ in heart failure, and to the best of our knowledge, only two other reports of the association between ‘self‐efficacy’ and fatal and non‐fatal outcomes in large cohorts have been published. The revised European Heart Failure Self‐care Behaviour scale (EHFScB‐9) is one such instrument with nine questions covering daily weighing; contacting a doctor or nurse for worsening dyspnoea, fatigue, oedema, or increasing weight (three questions); fluid and salt restriction (two questions); medication adherence; and regular exercise. Replies to each question are provided using a 5‐point Likert scale (ranging from ‘I completely agree’ to ‘I don't agree at all’). In a study of 559 consecutive patients with chronic heart failure from the Netherlands, poor self‐care behaviour, defined by a score below the median, was not associated with worse outcomes.^13^ However, in a more recent and larger study of 1123 consecutive Spanish patients with chronic heart failure and at least one recent hospitalization, patients with poor global self‐care (lowest tertile of EHFScB‐9 scores, <55/100 points, *n* = 349) had a higher relative risk of all‐cause death (HR 1.29, 95% CI 1.07–1.55; *p* = 0.007), consistent with the present study (HR 1.18, 95% CI 1.08–1.28 and HR 1.21, 95% CI 1.12–1.32, for ‘poor’ vs. ‘good’ knowledge regarding ‘prevention’ and ‘response’ question, respectively).^14^

Clearly, the key question raised by this Spanish study and our analyses is whether self‐care behaviours can be improved and whether such improvement is demonstrated to translate into better outcomes. This question is crucial given that our results show that nearly a quarter of our patients reported ‘poor’ knowledge of self‐care, showing there is a big deficit in this aspect of management. A recent study found that patients have low self‐confidence, in particular, related to regular exercising, eating a low‐salt diet, and flu vaccination.^15^ Various approaches to teaching self‐care behaviours have been described, but, of concern, physicians appear to engage poorly with this aspect of patient management.^16^, ^17^, ^18^, ^19^, ^20^, ^21^, ^22^ Newer approaches may be valuable, as suggested by the Swedish Self‐care Management Intervention in Heart Failure (SMART‐HF) multicentre randomized trial. Although small (*n* = 118), this demonstrated that a mobile device‐based educational intervention improved the EHFScB‐9 score compared with usual care and resulted in fewer days spent in hospital.^23^ Our findings suggest that the KCCQ ‘self‐efficacy’ questions may help identify patients who could benefit from educational interventions of this type and there is recent evidence that specialist nurses can successfully deliver such interventions.^24^, ^25^

### Limitations

The patients enrolled in the clinical trials analysed were relatively selected and, therefore, may not fully represent ‘real‐world’ patients. We had no additional information on any formal assessment of patient knowledge about self‐care, what education might have been provided to patients, or whether self‐reported knowledge translates into altered health behaviours. Patients enrolled in trials may also be more adherent to therapy than the general population and as part of the trial protocol may have more access to advice and care. Finally, we did not have information on patient socioeconomic status or education level which may also have influenced their level of self‐knowledge and outcomes.

## Conclusions

Poor self‐reported knowledge about ‘prevention’ and ‘response’ strategies, evaluated by the KCCQ, was associated with a worse heart failure clinical profile, and a higher risk of cardiovascular and overall mortality. Evaluating knowledge of ‘self‐efficacy’ may provide prognostic information and a guide to which patients may benefit further from education about self‐management.

### Funding

J.J.V.M. and P.S.J. are supported by a British Heart Foundation Centre of Research Excellence Grant RE/18/6/34217 and the Vera Melrose Heart Failure Research Fund. M.Y. is funded by the China Scholarship Council.

**Conflict of interest**: none declared.

## Supporting information


**Appendix S1.** Supporting Information.
