# Supplementary material for: Knowledge about self‐efficacy and outcomes in patients with heart failure and reduced ejection fraction
Source: Eur J Heart Fail. 2023 Jul 26;25(10):1831–9. doi: 10.1002/ejhf.2944 (PMC10947165; doi:10.1002/ejhf.2944)

**Knowledge about self-efficacy and outcomes in patients with heart failure and reduced ejection fraction**

Supplemental material

| **Tables** | **Page** |
| --- | --- |
| Supplemental Table 1 | 3 |
| Supplemental Table 2 | 6 |
| Supplemental Table 3 | 7 |
| Supplemental Table 4 | 8 |
| Supplemental Table 5 | 9 |
| Supplemental Table 6 | 14 |
| Supplemental Table 7 | 17 |
| Supplemental Table 8 | 20 |
| **Figures** |  |
| Supplemental Figure 1 | 26 |
| Supplemental Figure 2 | 27 |
| Supplemental Figure 3 | 28 |
| Supplemental Figure 4 | 29 |

**Tables**

Table1: Summary of the HFrEF trials

Table 2: KCCQ question 10 (response) and 11 (prevention), and choice of answers

Table 3: Patients with answers for KCCQ question 10 (response) and 11 (prevention) in each trial

Table 4: Patients completed KCCQ question 10 (response) and 11 (prevention) according to answers in each trial

Table 5: Baseline characteristics between non-responders and responders of KCCQ self-care questions in patients with HFrEF

Table 6: Clinical outcomes of patients with poor and good KCCQ self-rated knowledge and HFrEF

Table 7: Clinical outcomes of non-responders and responders of KCCQ self-care questions in patients with HFrEF

**Figures**

Figure 1: Distribution of the patients according to the category of KCCQ question 10 (response) and 11 (prevention)

Figure 2: Patients reporting to KCCQ question 10 (response) and 11 (prevention) according to sex

Figure 3: Patients reporting to KCCQ question 10 (response) and 11 (prevention) according to age

Figure 4: Patients reporting to KCCQ question 10 (response) and 11 (prevention) according to region

Table 1: Summary of the HFrEF trials

|  | **ATMOSPHERE** | **PARADIGM-HF** | **DAPA-HF** |
| --- | --- | --- | --- |
| **N** | 7016 | 8399 | 4744 |
| **Intervention** | Aliskiren/enalapril  Aliskiren 300mg OD | Sacubitril/valsartan  49/51 mg BID (LCZ696 200mg BID) | Dapagliflozin 10 mg OD |
| **Compared with** | Enalapril 5-10mg  BID | Enalapril 10mg  BID | Placebo |
| **Period** | 2009-2015 | 2009-2014 | 2017.02-2018.08 |
| **Duration** | Median 36.6 mo | Median 27 mo | Median 18.2 mo |
| **Sites** | 789 centers /43 countries | 1043 centers /47  countries | 410 centers /20 countries |
| **Core inclusion criteria** | Age≥18 yrs;  NYHA class: II-IV;  LVEF≤35% | Age≥18 yrs;  NYHA class: II-IV;  LVEF≤40%/35% (since December  2012) | Age≥18 yrs;  NYHA class: II-IV;  LVEF≤40%;  established DM or HbA1c ≥6.5% (48 mmol/mol) |
| **Prior HF hosp (HHF)**  **(BNP-pg/ml; NT-proBNP-pg/ml)** | If HHF within prior 12 mo, BNP ≥100 or NT-proBNP ≥400; if not, BNP ≥150 or  NT-proBNP ≥600 | If HHF within prior 12 mo, BNP ≥100 or NT-proBNP ≥400; if not, BNP ≥150 or NT-proBNP ≥600 | If HHF within prior 12 mo, NT-proBNP ≥400; if not, BNP ≥150 or NT-proBNP ≥600; if atrial fibrillation/flutter on baseline ECG, NT-proBNP ≥900 |
| **Key exclusion criteria**  **(BP-mm Hg; Potassium-mmol/L;**  **Serum Cr-umol/L;**  **eGFR-ml/min/1.73m^2^)** | Symptomatic hypotension; SBP  <95 at visit 1 or <90 at randomization; potassium ≥5.0 at screening, or ≥5.2 at randomization; eGFR <40 at screening, or <35 at randomization, or decrease >25% from screening to randomization | Symptomatic hypotension; SBP <100 at screening or <95 at randomisation; potassium >5.2 at screening, or >5.4  at randomization; eGFR <30 at screening, or <35 at randomization | Symptoms of hypotension or a SBP of < 95 mmHg; eGFR < 30 (or rapidly declining renal function) |
| **Primary Outcomes** | 1^st^ occurrence of either CVD  or HHF | 1^st^ occurrence of either CVD or HHF | Worsening HF (hosp or urgent visit resulting in i.v. therapy) or CVD |

ATOMOSPHERE = Aliskiren Trial to Minimize Outcomes in Patients with Heart Failure; PARADIGM-HF = Prospective Comparison of ARNI With ACEI to Determine Impact on Global Mortality and Morbidity in Heart Failure; DAPA-HF = Dapagliflozin And Prevention of Adverse outcomes in Heart Failure; HFrEF, heart failure with reduced ejection fraction.

BID, twice a day; BNP, B-type natriuretic peptide; Cr, creatinine; CVD, cardiovascular death; DM, diabetes mellitus; ECG, electrocardiogram; eGFR, estimated glomerular filtration rate; HbA1c, hemoglobin A1c; HHF, hospitalization for heart failure; HF, heart failure; hosp, hospitalization; i.v. intravenous; LVEF, left ventricular ejection fraction; mo, month; NT-proBNP, N-terminal pro B-type natriuretic peptide; NYHA, New York Heart Association; SBP, systolic blood pressure; OD, once daily; yr, year.

Table 2: KCCQ question 10 (response) and 11 (prevention), and choice of answers

| Q10 | Heart Failure symptoms can worsen for a number of reasons. How sure are you that you know what to do, or whom to call, if your heart failure gets worse? | | | | |
| --- | --- | --- | --- | --- | --- |
| □ Not at all sure | | □ Not very sure | □ Somewhat sure | □ Mostly sure | □ Completely sure |
| Q11 | How well do you understand what things you are able to do to keep your heart failure symptoms from getting worse? (for example, weighing yourself, eating a low salt diet, etc.) | | | | |
| □ Do not understand at all | | □ Do not understand very well | □ Somewhat understand | □ Mostly understand | □ Completely understand |

Q10, KCCQ question 10; Q11, KCCQ question 11; KCCQ, Kansas City Cardiomyopathy Questionnaire.

Table 3: Patients with answers for KCCQ question 10 (response) and 11 (prevention) in each trial

| **Question** | **ATMOSPHERE**  **N=7016** | **PARADIGM-HF**  **N=8399** | **DAPA-HF**  **N=4744** | **Total**  **N=20159** |
| --- | --- | --- | --- | --- |
| KCCQ question 10 (response) | 5925 (84.5) | 7611 (90.6) | 4101 (86.5) | 17637 (87.5) |
| KCCQ question 11 (prevention) | 5918 (84.4) | 7610 (90.6) | 4101 (86.5) | 17629 (87.4) |

Data are shown as N (%). PARADIGM-HF, Prospective Comparison of ARNI With ACEI to Determine Impact on Global Mortality and Morbidity in Heart Failure; PARAGON-HF, Prospective Comparison of ARNI with ARB Global Outcomes in HF with Preserved Ejection Fraction. KCCQ, Kansas City Cardiomyopathy Questionnaire.

Table 4: Patients completed KCCQ question 10 (response) and 11 (prevention) according to answers in each trial

| **Question** | **ATMOSPHERE**  **N=7016** | | | **PARADIGM-HF**  **N=8399** | | | **DAPA-HF**  **N=4744** | | |
| --- | --- | --- | --- | --- | --- | --- | --- | --- | --- |
| **Q10** |  |  |  |  |  |  |  |  |  |
|  | Poor  1407 (23.8) | Not at all | 122 (2.1) | Poor  1509 (19.8) | Not at all | 121 (1.6) | Poor  1117 (27.2) | Not at all | 130 (3.2) |
|  |  | Not very | 359 (6.1) |  | Not very | 337 (4.4) |  | Not very | 304 (7.4) |
|  |  | Somewhat | 926 (15.6) |  | Somewhat | 1051 (13.8) |  | Somewhat | 683 (16.7) |
|  | Fair | Mostly | 1907 (32.2) | Fair | Mostly | 2417 (31.8) | Fair | Mostly | 1139 (27.8) |
|  | Good | Completely | 2611 (44.1) | Good | Completely | 3685 (48.4) | Good | Completely | 1845 (45.0) |
| **Q11** |  |  |  |  |  |  |  |  |  |
|  | Poor  1539 (26.0) | Not at all | 61 (1.0) | Poor  1526 (20.1) | Not at all | 58 (0.8) | Poor  1132 (27.6) | Not at all | 61 (1.5) |
|  |  | Not very | 307 (5.2) |  | Not very | 270 (3.6) |  | Not very | 261 (6.4) |
|  |  | Somewhat | 1171 (19.8) |  | Somewhat | 1198 (15.7) |  | Somewhat | 810 (19.8) |
|  | Fair | Mostly | 2369 (40.0) | Fair | Mostly | 3091 (40.6) | Fair | Mostly | 1437 (35.0) |
|  | Good | Completely | 2010 (34.0) | Good | Completely | 2993 (39.3) | Good | Completely | 1532 (37.4) |

Data are shown as N (%). ATMOSPHERE, Aliskiren Trial to Minimize Outcomes in Patients with Heart Failure; PARADIGM-HF, Prospective Comparison of ARNI With ACEI to Determine Impact on Global Mortality and Morbidity in Heart Failure; DAPA-HF, Dapagliflozin And Prevention of Adverse outcomes in Heart Failure trial. Q10, KCCQ question 10 (response); Q11, KCCQ question 11 (prevention); KCCQ, Kansas City Cardiomyopathy Questionnaire.

Table 5: Baseline characteristics between non-responders and responders of KCCQ self-care questions in patients with HFrEF

|  | **KCCQ “prevention” question** | | | **KCCQ “response” question** | | |
| --- | --- | --- | --- | --- | --- | --- |
|  | **Non-responders** | **Responders** | **P value** | **Non-responders** | **Responders** | **P value** |
| N, (%) | 2530 (12.6) | 17629 (87.4) |  | 2522 (12.5) | 17637 (87.5) |  |
| **Demographic characteristics** |  |  |  |  |  |  |
| Age, yr | 62.1 ± 13.2 | 64.5 ± 11.2 | <0.001 | 62.1 ± 13.2 | 64.5 ± 11.2 | <0.001 |
| Age >70, yr | 732 (28.9) | 5674 (32.2) | <0.001 | 730 (28.9) | 5676 (32.2) | <0.001 |
| Gender |  |  | <0.001 |  |  | <0.001 |
| Female | 639 (25.3) | 3827 (21.7) |  | 638 (25.3) | 3828 (21.7) |  |
| Male | 1891 (74.7) | 13802 (78.3) |  | 1884 (74.7) | 13809 (78.3) |  |
| Region |  |  | <0.001 |  |  | <0.001 |
| North America | 36 (1.4) | 1420 (8.1) |  | 36 (1.4) | 1420 (8.1) |  |
| Latin America† | 465 (18.4) | 2904 (16.5) |  | 468 (18.6) | 2901 (16.4) |  |
| Western Europe | 147 (5.8) | 4305 (24.4) |  | 141 (5.6) | 4311 (24.4) |  |
| Eastern Europe†† | 125 (4.9) | 6249 (35.4) |  | 121 (4.8) | 6253 (35.5) |  |
| Asia/Pacific and other | 1757 (69.4) | 2751 (15.6) |  | 1756 (69.6) | 2752 (15.6) |  |
| Race |  |  | <0.001 |  |  | <0.001 |
| White | 344 (13.6) | 13125 (74.5) |  | 334 (13.2) | 13135 (74.5) |  |
| Black | 61 (2.4) | 702 (4.0) |  | 62 (2.5) | 701 (4.0) |  |
| Asian | 1709 (67.5) | 2680 (15.2) |  | 1708 (67.7) | 2681 (15.2) |  |
| Others | 416 (16.4) | 1122 (6.4) |  | 418 (16.6) | 1120 (6.4) |  |
| SBP, mmHg | 119.9 ± 17.1 | 122.6 ± 16.5 | <0.001 | 119.9 ± 17.1 | 122.6 ± 16.6 | <0.001 |
| SBP <110 mmHg | 713 (28.2) | 3705 (21.0) | <0.001 | 711 (28.2) | 3707 (21.0) | <0.001 |
| HR, bpm | 73.4 ± 12.5 | 71.7 ± 12.1 | <0.001 | 73.4 ± 12.5 | 71.7 ± 12.1 | <0.001 |
| BMI, kg/m^2^ | 24.6 (22.0-27.8) | 27.6 (24.5-31.2) | <0.001 | 24.5 (22.0-27.8) | 27.6 (24.5-31.2) | <0.001 |
| Weight category |  |  | <0.001 |  |  | <0.001 |
| <18.5 | 138 (5.5) | 256 (1.5) |  | 138 (5.5) | 256 (1.5) |  |
| 18.5-25.0 | 1193 (47.3) | 4633 (26.3) |  | 1192 (47.5) | 4634 (26.3) |  |
| 25.0-30 | 829 (32.9) | 6763 (38.4) |  | 825 (32.8) | 6767 (38.4) |  |
| ≥30.0 | 360 (14.3) | 5956 (33.8) |  | 357 (14.2) | 5959 (33.8) |  |
|  |  |  |  |  |  |  |
| **Comorbidities** |  |  |  |  |  |  |
| Atrial fibrillation (history) | 643 (25.4) | 6656 (37.8) | <0.001 | 639 (25.3) | 6660 (37.8) | <0.001 |
| Hypertension | 1437 (56.8) | 12358 (70.1) | <0.001 | 1432 (56.8) | 12363 (70.1) | <0.001 |
| Angina Pectoris | 453 (17.9) | 4682 (26.6) | <0.001 | 448 (17.8) | 4687 (26.6) | <0.001 |
| MI | 905 (35.8) | 7668 (43.5) | <0.001 | 903 (35.8) | 7670 (43.5) | <0.001 |
| Prior PCI/CABG | 660 (26.1) | 6065 (34.4) | <0.001 | 654 (25.9) | 6071 (34.4) | <0.001 |
| Stroke | 210 (8.3) | 1473 (8.4) | 0.93 | 210 (8.3) | 1473 (8.4) | 0.97 |
| COPD | 195 (7.7) | 2257 (12.8) | <0.001 | 195 (7.7) | 2257 (12.8) | <0.001 |
| Diabetes Mellitus | 836 (33.0) | 5998 (34.0) | 0.33 | 830 (32.9) | 6004 (34.0) | 0.26 |
| Anemia§ | 817 (32.6) | 3795 (21.9) | <0.001 | 816 (32.7) | 3796 (21.9) | <0.001 |
| Current smoker | 317 (12.5) | 2485 (14.1) | 0.033 | 314 (12.5) | 2488 (14.1) | 0.025 |
|  |  |  |  |  |  |  |
| **HF characteristics and investigations** |  |  |  |  |  |  |
| Ischemic etiology | 1388 (54.9) | 10252 (58.2) | 0.002 | 1381 (54.8) | 10259 (58.2) | 0.001 |
| Previous hospitalization for HF | 1416 (56.0) | 10297 (58.4) | 0.020 | 1412 (56.0) | 10301 (58.4) | 0.021 |
| NYHA III/IV | 600 (23.7) | 5597 (31.8) | <0.001 | 597 (23.7) | 5600 (31.8) | <0.001 |
| KCCQ clinical summary score | 85.0 ± 14.9 | 74.1 ± 19.9 | <0.001 | 85.1 ± 14.9 | 74.1 ± 19.9 | <0.001 |
| Signs of congestion |  |  |  |  |  |  |
| Dyspnea on effort | 1446 (76.8) | 11721 (86.7) | <0.001 | 1440 (76.8) | 11727 (86.7) | <0.001 |
| Dyspnea at rest | 49 (2.6) | 563 (4.2) | 0.001 | 49 (2.6) | 563 (4.2) | 0.001 |
| Orthopnea | 135 (7.2) | 836 (6.2) | 0.098 | 135 (7.2) | 836 (6.2) | 0.087 |
| PND | 68 (3.6) | 688 (5.1) | 0.005 | 68 (3.6) | 688 (5.1) | 0.006 |
| Fatigue | 655 (34.8) | 7036 (52.1) | <0.001 | 649 (34.7) | 7042 (52.1) | <0.001 |
| Edema | 287 (15.2) | 2905 (21.5) | <0.001 | 284 (15.2) | 2908 (21.5) | <0.001 |
| S3 gallop | 267 (14.2) | 1122 (8.3) | <0.001 | 268 (14.3) | 1121 (8.3) | <0.001 |
| JVD | 212 (11.3) | 1265 (9.4) | 0.009 | 211 (11.3) | 1266 (9.4) | 0.009 |
| Rales | 144 (7.7) | 1221 (9.0) | 0.048 | 144 (7.7) | 1221 (9.0) | 0.054 |
| ECG findings and NT-proBNP |  |  |  |  |  |  |
| Atrial fibrillation/flutter | 446 (17.7) | 4442 (25.4) | <0.001 | 442 (17.6) | 4446 (25.4) | <0.001 |
| NT-proBNP, pg/ml | 1430 (780-2854) | 1421 (792-2720) | 0.45 | 1430 (780-2848) | 1420 (792-2721) | 0.43 |
| Atrial fibrillation/flutter§§ | 1840 (1075-3191) | 1867 (1144-3243) | 0.36 | 1840 (1075-3191) | 1867 (1144-3243) | 0.37 |
| No atrial fibrillation/flutter§§ | 1355 (728-2783) | 1264 (719-2471) | 0.006 | 1358 (729-2783) | 1264 (719-2471) | 0.005 |
| LVEF and other laboratory investigations |  |  |  |  |  |  |
| LVEF, % | 28.5 ± 6.3 | 29.6 ± 6.2 | <0.001 | 28.5 ± 6.3 | 29.6 ± 6.2 | <0.001 |
| Hemoglobin, g/L | 134.0 (122.0-146.0) | 139.0 (128.0-149.0) | <0.001 | 134.0 (122.0-146.0) | 139.0 (128.0-149.0) | <0.001 |
| Creatinine, μmol/L | 91.1 (76.0-110.0) | 94.0 (80.0-112.0) | <0.001 | 91.1 (76.0-110.0) | 94.0 (80.0-112.0) | <0.001 |
| eGFR, mL/min/1.73m^2^ | 70.0 (55.0-84.0) | 68.0 (54.0-82.0) | <0.001 | 70.0 (55.0-84.0) | 68.0 (54.0-82.0) | <0.001 |
| eGFR<60, mL/min/1.73m^2^ | 793 (31.3) | 6043 (34.3) | 0.003 | 792 (31.4) | 6044 (34.3) | 0.004 |
|  |  |  |  |  |  |  |
| **Medication and other interventions** |  |  |  |  |  |  |
| Diuretics | 2032 (80.3) | 14737 (83.6) | <0.001 | 2025 (80.3) | 14744 (83.6) | <0.001 |
| Loop | 1907 (75.4) | 13384 (75.9) | 0.55 | 1901 (75.4) | 13390 (75.9) | 0.55 |
| Thiazides | 118 (4.7) | 1273 (7.2) | <0.001 | 117 (4.6) | 1274 (7.2) | <0.001 |
| Digitalis | 892 (35.3) | 4776 (27.1) | <0.001 | 890 (35.3) | 4778 (27.1) | <0.001 |
| Beta-blocker | 2227 (88.0) | 16574 (94.0) | <0.001 | 2220 (88.0) | 16581 (94.0) | <0.001 |
| MRA | 1072 (42.4) | 9571 (54.3) | <0.001 | 1070 (42.4) | 9573 (54.3) | <0.001 |
| ACEI/ARB/ARNI | 2466 (97.5) | 17391 (98.6) | <0.001 | 2458 (97.5) | 17399 (98.7) | <0.001 |
| ICD## | 185 (7.3) | 3348 (19.0) | <0.001 | 182 (7.2) | 3351 (19.0) | <0.001 |
| CRT-P or CRT-D | 85 (3.4) | 1236 (7.0) | <0.001 | 86 (3.4) | 1235 (7.0) | <0.001 |

Data are presented as mean ± SD, median (IQR) for continuous measures, and n (%) for categorical measures.

Abbreviations and definitions as in Table 1.

Table 6: Clinical outcomes of patients with poor and good KCCQ self-rated knowledge and HFrEF

|  | **Both KCCQ questions** | **Both KCCQ questions** |
| --- | --- | --- |
|  | **Poor** | **Good** |
| N (%) | 2590 (33.3) | 5185 (66.7) |
| **Primary composite outcome** |  |  |
| No. of events (%) | 679 (26.2) | 1276 (24.6) |
| Event rate per 100 person-years (95% CI) | 12.59 (11.67-13.57) | 11.42 (10.81-12.06) |
| Unadjusted HR (95% CI) | 1.00 (Ref.) | 0.91 (0.83-1.00) |
| P value |  | 0.042 |
| Adjusted HR (95% CI)* | 1.00 (Ref.) | 0.96 (0.87-1.05) |
| P value |  | 0.370 |
| **First HF hosp.** |  |  |
| No. of events (%) | 363 (14.0) | 776 (15.0) |
| Event rate per 100 person-years (95% CI) | 6.73 (6.07-7.46) | 6.94 (6.47-7.45) |
| Unadjusted HR (95% CI) | 1.00 (Ref.) | 1.03 (0.91-1.17) |
| P value |  | 0.607 |
| Adjusted HR (95% CI)* | 1.00 (Ref.) | 1.02 (0.90-1.17) |
| P value |  | 0.734 |
| **CV death** |  |  |
| No. of events (%) | 459 (17.7) | 771 (14.9) |
| Event rate per 100 person-years (95% CI) | 7.89 (7.20-8.65) | 6.40 (5.97-6.87) |
| Unadjusted HR (95% CI) | 1.00 (Ref.) | 0.81 (0.72-0.91) |
| P value |  | <0.001 |
| Adjusted HR (95% CI)* | 1.00 (Ref.) | 0.89 (0.79-1.01) |
| P value |  | 0.063 |
| **All-cause death** |  |  |
| No. of events (%) | 557 (21.5) | 935 (18.0) |
| Event rate per 100 person-years (95% CI) | 9.58 (8.81-10.41) | 7.76 (7.28-8.28) |
| Unadjusted HR (95% CI) | 1.00 (Ref.) | 0.81 (0.73-0.90) |
| P value |  | <0.001 |
| Adjusted HR (95% CI)* | 1.00 (Ref.) | 0.87 (0.78-0.98) |
| P value |  | 0.017 |
| **Recurrent HF hosp./CV death** |  |  |
| No. of events | 1042 | 2067 |
| Event rate per 100 person-years (95% CI) | 17.91 (16.41-19.55) | 17.16 (16.02-18.39) |
| Unadjusted IRR (95% CI) | 1.00 (Ref.) | 0.89 (0.78-1.01) |
| P value |  | 0.066 |
| Adjusted IRR (95% CI)* | 1.00 (Ref.) | 0.95 (0.84-1.07) |
| P value |  | 0.403 |

Abbreviations and definitions as in Table 2.

Table 7: Clinical outcomes of non-responders and responders of KCCQ self-care questions in patients with HFrEF

|  | **KCCQ “prevention” question** | | **KCCQ “response” question** | |
| --- | --- | --- | --- | --- |
|  | **Non-responders** | **Responders** | **Non-responders** | **Responders** |
| N (%) | 2530 (12.6) | 17629 (87.4) | 2522 (12.5) | 17637 (87.5) |
| **Primary composite outcome** |  |  |  |  |
| No. of events (%) | 699 (27.6) | 4578 (26.0) | 696 (27.6) | 4581 (26.0) |
| Event rate per 100 person-years (95% CI) | 13.02 (12.09-14.02) | 12.06 (11.71-12.41) | 13.01 (12.08-14.01) | 12.06 (11.71-12.41) |
| Unadjusted HR (95% CI) | 1.00 (Ref.) | 0.92 (0.85-1.00) | 1.00 (Ref.) | 0.93 (0.85-1.00) |
| P value |  | 0.054 |  | 0.057 |
| Adjusted HR (95% CI)* | 1.00 (Ref.) | 0.95 (0.87-1.04) | 1.00 (Ref.) | 0.95 (0.87-1.04) |
| P value |  | 0.256 |  | 0.310 |
| **First HF hosp.** |  |  |  |  |
| No. of events (%) | 376 (14.9) | 2692 (15.3) | 375 (14.9) | 2693 (15.3) |
| Event rate per 100 person-years (95% CI) | 7.00 (6.33-7.75) | 7.09 (6.83-7.36) | 7.01 (6.33-7.76) | 7.09 (6.83-7.36) |
| Unadjusted HR (95% CI) | 1.00 (Ref.) | 1.01 (0.91-1.12) | 1.00 (Ref.) | 1.01 (0.91-1.12) |
| P value |  | 0.863 |  | 0.875 |
| Adjusted HR (95% CI)* | 1.00 (Ref.) | 0.97 (0.86-1.10) | 1.00 (Ref.) | 0.97 (0.86-1.10) |
| P value |  | 0.614 |  | 0.636 |
| **CV death** |  |  |  |  |
| No. of events (%) | 469 (18.5) | 2903 (16.5) | 467 (18.5) | 2905 (16.5) |
| Event rate per 100 person-years (95% CI) | 8.06 (7.37-8.83) | 7.05 (6.80-7.32) | 8.06 (7.36-8.82) | 7.06 (6.80-7.32) |
| Unadjusted HR (95% CI) | 1.00 (Ref.) | 0.88 (0.79-0.96) | 1.00 (Ref.) | 0.88 (0.79-0.97) |
| P value |  | 0.007 |  | 0.008 |
| Adjusted HR (95% CI)* | 1.00 (Ref.) | 0.96 (0.86-1.08) | 1.00 (Ref.) | 0.97 (0.87-1.09) |
| P value |  | 0.523 |  | 0.611 |
| **All-cause death** |  |  |  |  |
| No. of events (%) | 538 (21.3) | 3508 (19.9) | 536 (21.3) | 3510 (19.9) |
| Event rate per 100 person-years (95% CI) | 9.25 (8.50-10.06) | 8.52 (8.25-8.81) | 9.24 (8.49-10.06) | 8.52 (8.25-8.81) |
| Unadjusted HR (95% CI) | 1.00 (Ref.) | 0.92 (0.84-1.01) | 1.00 (Ref.) | 0.92 (0.84-1.01) |
| P value |  | 0.080 |  | 0.082 |
| Adjusted HR (95% CI)* | 1.00 (Ref.) | 0.95 (0.86-1.06) | 1.00 (Ref.) | 0.96 (0.86-1.06) |
| P value |  | 0.351 |  | 0.416 |
| **Recurrent HF hosp./CV death** |  |  |  |  |
| No. of events | 1088 | 7412 | 1084 | 7416 |
| Event rate per 100 person-years (95% CI) | 18.70 (17.16-20.39) | 18.01 (17.39-18.65) | 18.70 (17.15-20.38) | 18.01 (17.39-18.65) |
| Unadjusted IRR (95% CI) | 1.00 (Ref.) | 0.90 (0.81-1.01) | 1.00 (Ref.) | 0.90 (0.81-1.01) |
| P value |  | 0.078 |  | 0.077 |
| Adjusted IRR (95% CI)* | 1.00 (Ref.) | 0.99 (0.88-1.11) | 1.00 (Ref.) | 0.99 (0.88-1.11) |
| P value |  | 0.851 |  | 0.815 |

Abbreviations and definitions as in Table 2.

Table 8a: Clinical outcomes according to KCCQ self-efficacy domain score in patients with HFrEF

|  | **KCCQ self-efficacy domain score** | | | | |
| --- | --- | --- | --- | --- | --- |
|  | $\boldsymbol{\geq}$**0,** $\boldsymbol{\leq}$**50** | **62.5** | **75** | **87.5** | **100** |
| N (%) | 3151 (17.9) | 1800 (10.2) | 4218 (23.9) | 3281 (18.6) | 5193 (29.4) |
| **Primary composite outcome** |  |  |  |  |  |
| No. of events (%) | 826 (26.2) | 523 (29.1) | 1103 (26.2) | 851 (25.9) | 1279 (24.6) |
| Event rate per 100 person-years (95% CI) | 12.63  (11.79-13.52) | 13.78  (12.65-15.01) | 12.03  (11.34-12.77) | 11.65  (10.89-12.46) | 11.42  (10.81-12.06) |
| Unadjusted HR (95% CI) | 1.00 (Ref.) | 1.09 (0.98-1.22) | 0.96 (0.87-1.05) | 0.93 (0.84-1.02) | 0.91 (0.83-0.99) |
| P value |  | 0.109 | 0.327 | 0.130 | 0.027 |
| Adjusted HR (95% CI)* | 1.00 (Ref.) | 1.10 (0.99-1.23) | 1.00 (0.92-1.10) | 0.97 (0.88-1.06) | 0.97 (088-1.06) |
| P value |  | 0.086 | 0.962 | 0.479 | 0.471 |
| **First HF hosp.** |  |  |  |  |  |
| No. of events (%) | 454 (14.4) | 314 (17.4) | 634 (15.0) | 515 (15.7) | 777 (15.0) |
| Event rate per 100 person-years (95% CI) | 6.94 (6.33-7.61) | 8.27 (7.41-9.24) | 6.92 (6.40-7.48) | 7.05 (6.47-7.69) | 6.94 (6.46-7.44) |
| Unadjusted HR (95% CI) | 1.00 (Ref.) | 1.20 (1.04-1.38) | 1.00 (0.89-1.13) | 1.03 (0.90-1.16) | 1.00 (0.89-1.13) |
| P value |  | 0.015 | 0.983 | 0.683 | 0.963 |
| Adjusted HR (95% CI)* | 1.00 (Ref.) | 1.17 (1.01-1.35) | 1.04 (0.92-1.18) | 1.03 (0.90-1.17) | 1.03 (0.92-1.16) |
| P value |  | 0.034 | 0.518 | 0.679 | 0.615 |
| **CV death** |  |  |  |  |  |
| No. of events (%) | 547 (17.4) | 331 (18.4) | 716 (17.0) | 538 (16.4) | 774 (14.9) |
| Event rate per 100 person-years (95% CI) | 7.71 (7.09-8.38) | 7.96 (7.15-8.87) | 7.20 (6.69-7.74) | 6.79 (6.24-7.39) | 6.41 (5.98-6.88) |
| Unadjusted HR (95% CI) | 1.00 (Ref.) | 1.03 (0.90-1.18) | 0.93 (0.83-1.04) | 0.88 (0.78-0.99) | 0.83 (0.74-0.93) |
| P value |  | 0.653 | 0.207 | 0.030 | 0.001 |
| Adjusted HR (95% CI)* | 1.00 (Ref.) | 1.06 (0.92-1.21) | 0.98 (0.88-1.10) | 0.94 (0.83-1.06) | 0.91 (0.82-1.02) |
| P value |  | 0.437 | 0.725 | 0.298 | 0.118 |
| **All-cause death** |  |  |  |  |  |
| No. of events (%) | 660 (21.0) | 410 (22.8) | 847 (20.1) | 656 (20.0) | 938 (18.1) |
| Event rate per 100 person-years (95% CI) | 9.30 (8.62-10.04) | 9.86 (8.95-10.87) | 8.51 (7.96-9.10) | 8.28 (7.67-8.94) | 7.77 (7.29-8.29) |
| Unadjusted HR (95% CI) | 1.00 (Ref.) | 1.06 (0.94-1.20) | 0.91 (0.83-1.01) | 0.89 (0.79-0.99) | 0.83 (0.76-0.92) |
| P value |  | 0.367 | 0.076 | 0.027 | <0.001 |
| Adjusted HR (95% CI)* | 1.00 (Ref.) | 1.07 (0.95-1.21) | 0.95 (0.86-1.05) | 0.93 (0.84-1.04) | 0.90 (0.81-0.99) |
| P value |  | 0.273 | 0.340 | 0.218 | 0.037 |
| **Recurrent HF hosp./CV death** |  |  |  |  |  |
| No. of events | 1287 | 863 | 1769 | 1428 | 2071 |
| Event rate per 100 person-years (95% CI) | 18.14  (16.75-19.64) | 20.77  (18.78-22.96) | 17.78  (16.58-19.07) | 18.02  (16.64-19.52) | 17.16  (16.02-18.39) |
| Unadjusted IRR (95% CI) | 1.00 (Ref.) | 1.13 (0.97-1.32) | 0.91 (0.80-1.03) | 0.94 (0.82-1.07) | 0.89 (0.79-1.00) |
| P value |  | 0.110 | 0.129 | 0.324 | 0.046 |
| Adjusted IRR (95% CI)* | 1.00 (Ref.) | 1.11 (0.96-1.28) | 0.97 (0.86-1.08) | 0.94 (0.83-1.06) | 0.96 (0.86-1.07) |
| P value |  | 0.155 | 0.547 | 0.282 | 0.442 |

Abbreviations and definitions as in Table 2.

Table 8b: Clinical outcomes according to KCCQ self-efficacy domain score in patients with HFrEF

|  | **KCCQ self-efficacy domain score** | | |
| --- | --- | --- | --- |
|  | **Poor (**$\boldsymbol{\geq}$**0,** $\boldsymbol{\leq}$**50)** | **Fair (>50,** $\boldsymbol{\leq}$**75)** | **Good (>75,** $\boldsymbol{\leq}$**100)** |
| N (%) | 3151 (17.9) | 6018 (34.1) | 8474 (48.0) |
| **Primary composite outcome** |  |  |  |
| No. of events (%) | 826 (26.2) | 1626 (27.0) | 2130 (25.1) |
| Event rate per 100 person-years (95% CI) | 12.63 (11.79-13.52) | 12.54 (11.95-13.17) | 11.51 (11.03-12.01) |
| Unadjusted HR (95% CI) | 1.00 (Ref.) | 1.00 (0.92-1.08) | 0.91 (0.84-0.99) |
| P value |  | 0.931 | 0.030 |
| Adjusted HR (95% CI)* | 1.00 (Ref.) | 1.03 (0.95-1.122) | 0.97 (0.89-1.05) |
| P value |  | 0.461 | 0.426 |
| **First HF hosp.** |  |  |  |
| No. of events (%) | 454 (14.4) | 948 (15.8) | 1292 (15.3) |
| Event rate per 100 person-years (95% CI) | 6.94 (6.33-7.61) | 7.31 (6.86-7.79) | 6.98 (6.61-7.37) |
| Unadjusted HR (95% CI) | 1.00 (Ref.) | 1.06 (0.95-1.18) | 1.01 (0.91-1.13) |
| P value |  | 0.320 | 0.825 |
| Adjusted HR (95% CI)* | 1.00 (Ref.) | 1.08 (0.97-1.21) | 1.03 (0.92-1.15) |
| P value |  | 0.178 | 0.597 |
| **CV death** |  |  |  |
| No. of events (%) | 547 (17.4) | 1047 (17.4) | 1312 (15.5) |
| Event rate per 100 person-years (95% CI) | 7.71 (7.09-8.38) | 7.42 (6.99-7.89) | 6.56 (6.22-6.93) |
| Unadjusted HR (95% CI) | 1.00 (Ref.) | 0.96 (0.87-1.07) | 0.85 (0.77-0.94) |
| P value |  | 0.446 | 0.001 |
| Adjusted HR (95% CI)* | 1.00 (Ref.) | 1.00 (0.90-1.11) | 0.92 (0.83-1.02) |
| P value |  | 0.955 | 0.131 |
| **All-cause death** |  |  |  |
| No. of events (%) | 660 (21.0) | 1257 (20.9) | 1594 (18.8) |
| Event rate per 100 person-years (95% CI) | 9.30 (8.62-10.04) | 8.91 (8.43-9.42) | 7.97 (7.59-8.37) |
| Unadjusted HR (95% CI) | 1.00 (Ref.) | 0.96 (0.87-1.05) | 0.85 (0.78-0.94) |
| P value |  | 0.339 | 0.001 |
| Adjusted HR (95% CI)* | 1.00 (Ref.) | 0.99 (0.90-1.09) | 0.91 (0.83-1.00) |
| P value |  | 0.801 | 0.054 |
| **Recurrent HF hosp./CV death** |  |  |  |
| No. of events | 1287 | 2632 | 3499 |
| Event rate per 100 person-years (95% CI) | 18.14 (16.75-19.64) | 18.66 (17.62-19.76) | 17.50 (16.61-18.44) |
| Unadjusted IRR (95% CI) | 1.00 (Ref.) | 0.97 (0.87-1.09) | 0.91 (0.81-1.01) |
| P value |  | 0.647 | 0.077 |
| Adjusted IRR (95% CI)* | 1.00 (Ref.) | 1.01 (0.91-1.12) | 0.95 (0.86-1.05) |
| P value |  | 0.885 | 0.321 |

Abbreviations and definitions as in Table 2.

Figure 1: Distribution of the patients according to the category of KCCQ question 10 (response) and 11 (prevention). Data are presented as percentage.





Figure 2: Patients reporting to KCCQ question 10 (response) and 11 (prevention) according to sex. Data are presented as percentage.


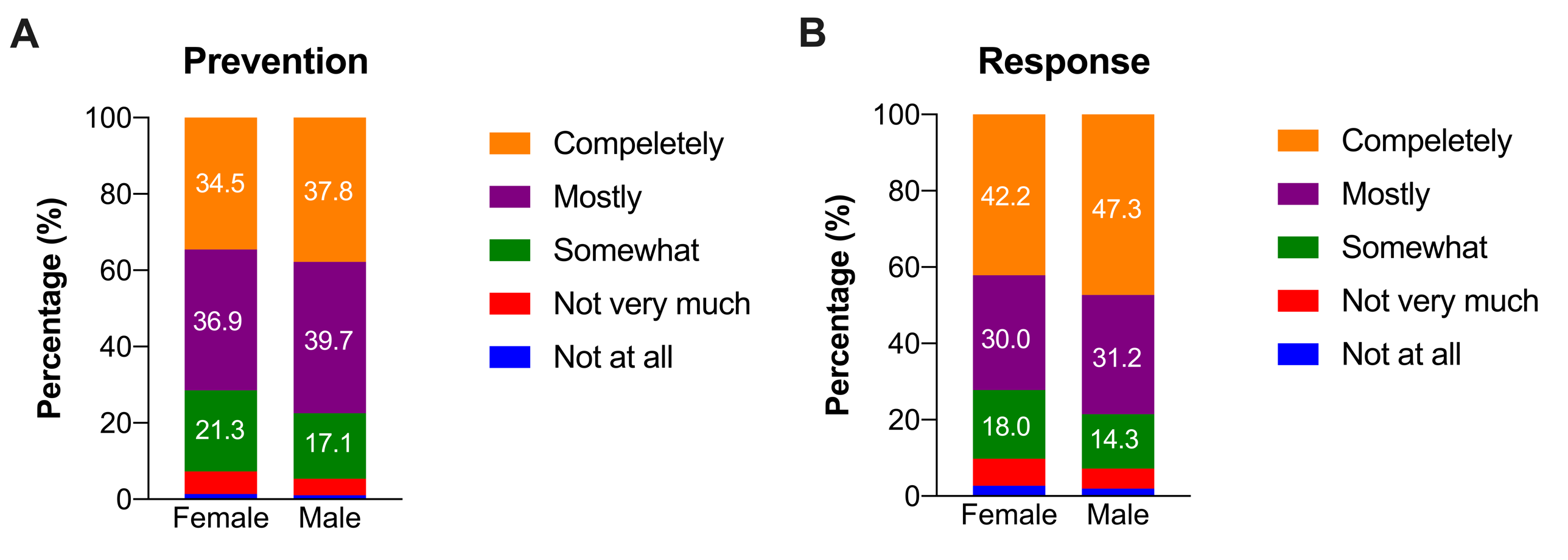


Figure 3: Patients reporting to KCCQ question 10 (response) and 11 (prevention) according to age. Data are presented as percentage.


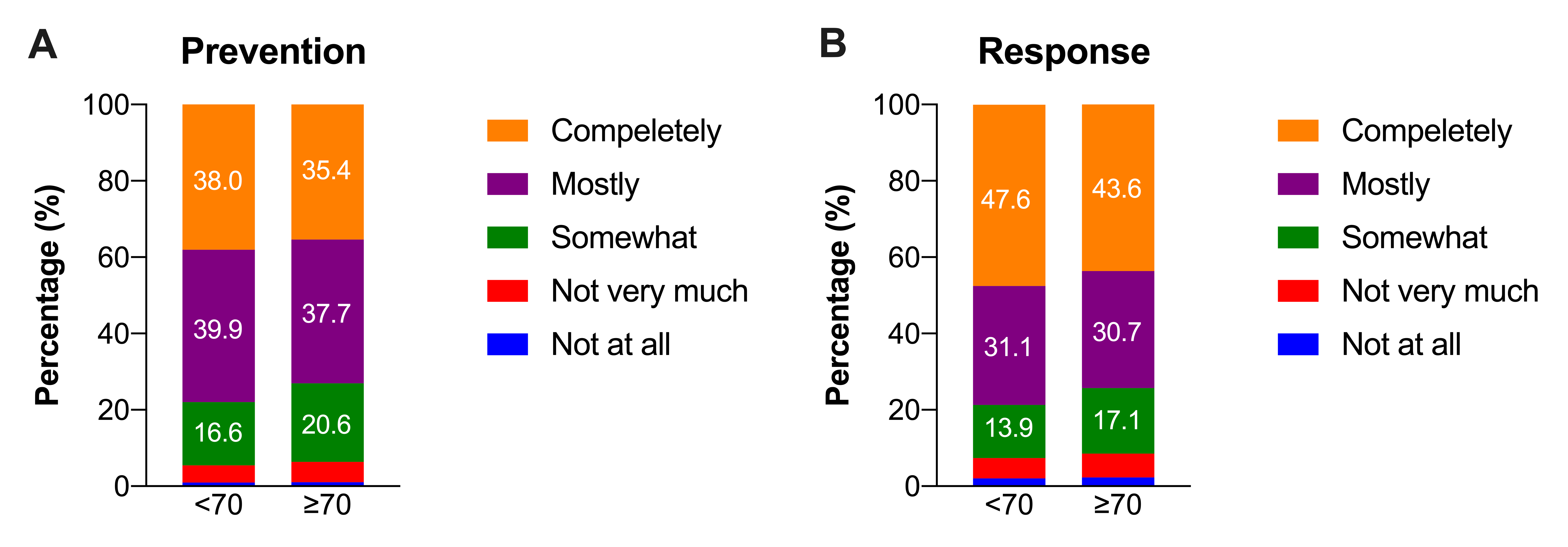


Figure 4: Patients reporting to KCCQ question 10 (response) and 11 (prevention) according to region. Data are presented as percentage.

NA, North America; LA, Latin America; WE, West Europe; CEER, Central/Eastern Europe and Russia; AP, Asia-Pacific.


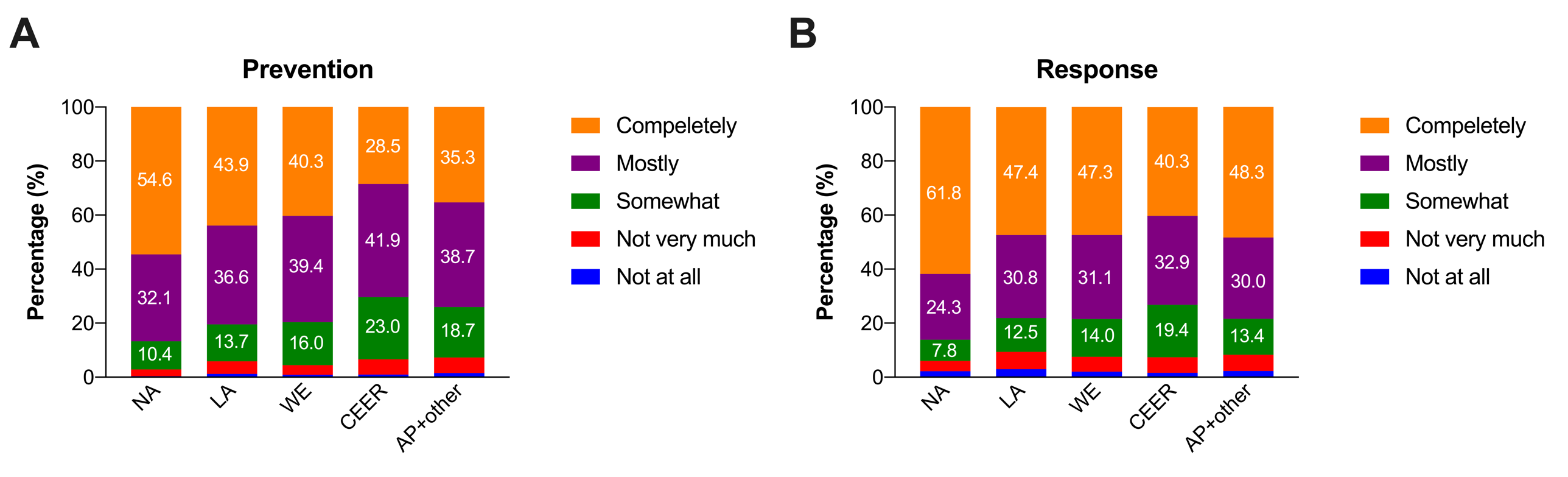

Supplement: Supplementary file 1 — Appendix S1. Supporting Information. [file EJHF-25-1831-s001.docx]
